# 11β,17aα-Dihydr­oxy-17aβ-methyl-d-homoandrosta-1,4-diene-3,17-dione monohydrate

**DOI:** 10.1107/S1600536809010770

**Published:** 2009-03-31

**Authors:** Ya Qiu, Ying Chen, Peng Xia

**Affiliations:** a Department of Medicinal Chemistry, School of Pharmacy, Fudan University, 138 Yixueyuan Road, Shanghai 200032, People’s Republic of China

## Abstract

In the title compound, C_21_H_28_O_4_·H_2_O, the cyclo­hexa­dienone ring is planar (r.m.s. deviation 0.0186 Å), whereas the two cyclo­hexane rings and the cyclo­hexa­none ring adopt chair conformations. The crystal structure is stabilized by O—H⋯O and C—H⋯O hydrogen bonds.

## Related literature

For general background, see: Conrow (1999 ▶). For details of the synthesis, see: Huo (2003 ▶).
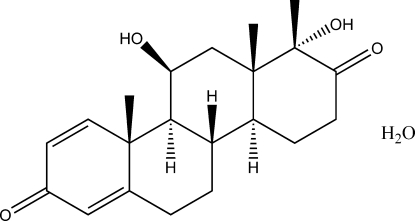


## Experimental

### 

#### Crystal data

C_21_H_28_O_4_·H_2_O
*M* *_r_* = 362.45
Monoclinic, 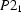

*a* = 6.641 (2) Å
*b* = 18.642 (6) Å
*c* = 8.017 (3) Å
β = 103.797 (4)°
*V* = 963.9 (6) Å^3^
*Z* = 2
Mo *K*α radiation
μ = 0.09 mm^−1^
*T* = 293 K
0.25 × 0.20 × 0.15 mm

#### Data collection

Bruker SMART APEX CCD area-detector diffractometer
Absorption correction: multi-scan (*SADABS*; Bruker, 2000 ▶) *T*_min_ = 0.978, *T*_max_ = 0.987
4866 measured reflections
2182 independent reflections
1778 reflections with *I* > 2σ(*I*)
*R*_int_ = 0.034

#### Refinement

*R*[*F* ^2^ > 2σ(*F* ^2^)] = 0.042
*wR*(*F* ^2^) = 0.104
*S* = 0.97
2182 reflections
254 parameters
5 restraints
H atoms treated by a mixture of independent and constrained refinement
Δρ_max_ = 0.24 e Å^−3^
Δρ_min_ = −0.15 e Å^−3^

### 

Data collection: *SMART* (Bruker, 2000 ▶); cell refinement: *SAINT* (Bruker, 2000 ▶); data reduction: *SAINT*; program(s) used to solve structure: *SHELXS97* (Sheldrick, 2008 ▶); program(s) used to refine structure: *SHELXL97* (Sheldrick, 2008 ▶); molecular graphics: *SHELXTL* (Sheldrick, 2008*b* ▶); software used to prepare material for publication: *SHELXTL*.

## Supplementary Material

Crystal structure: contains datablocks global, I. DOI: 10.1107/S1600536809010770/gk2193sup1.cif

Structure factors: contains datablocks I. DOI: 10.1107/S1600536809010770/gk2193Isup2.hkl

Additional supplementary materials:  crystallographic information; 3D view; checkCIF report

## Figures and Tables

**Table 1:** Hydrogen-bond geometry (Å, °)

*D*—H⋯*A*	*D*—H	H⋯*A*	*D*⋯*A*	*D*—H⋯*A*
O2—H2*X*⋯O5	0.821 (19)	2.00 (2)	2.799 (3)	166 (3)
O4—H4*X*⋯O1^i^	0.810 (18)	1.97 (2)	2.758 (3)	164 (3)
O5—H5*Y*⋯O3^ii^	0.86 (2)	1.99 (2)	2.851 (4)	171 (4)
O5—H5*X*⋯O4^iii^	0.85 (2)	1.97 (2)	2.811 (3)	172 (4)

Symmetry codes: (i) 
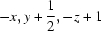
; (ii) 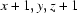; (iii) 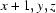.

**Fig. 1.**
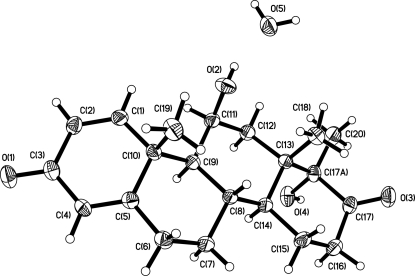
The molecular structure of the title compound showing displacement ellipsoids at the 30% probability level.

**Fig. 2.**
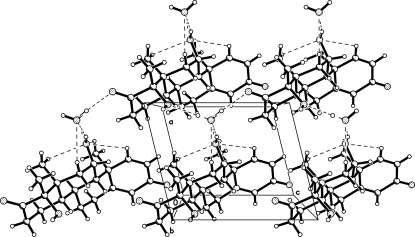
Crystal packing of the title compound - projection along the b axis.

## supplementary crystallographic information

### Comment

21-Methyl-11β,17α-dihydroxy-1,4-pregnadien-3,20-dione derivatives are intermediates in the synthesis of steroid agents (Conrow, 1999). We tried to prepare 21-methyl-11β,17α-dihydroxy-1,4-pregnadien-3,20-dione by 21-methylation of 11β,17α-dihydroxy-21-chloro-1,4-pregnadien-3,20-dione with organic metal reagent. However, the reaction of 11β,17α-dihydroxy-21-chloro-1,4-pregnadien-3,20-dione with a mixture of iodomethane, zinc dust, I_2_(cat.) and Cl_2_Ni(PPh_3_)_2_(cat.) in the solvent DMA offered the title compound as one of the main products. The crystal structure determination of the title compound was carried out in order to determine the exact molecular structure.

The molecular structure of the title compound is shown in Fig.1. The cyclohexadienone ring is planar. The two cyclohexane rings and the cyclohexanone ring adopt chair conformations. Intra- and intermolecular C—H···O and O—H···O hydrogen bonding are observed in the crystal structure.

### Experimental

A mixture of CH_3_I (568 mg, 4 mmol) (Huo,2003), zinc dust (387 mg, 6 mmol) and I_2 _(50 mg, 0.2 mmol) in dry DMA was stirred under N_2_ at 5°C until the red color of I_2_ disappeared. After stirring further at 30°C for 3 h, the11β,17α-dihydroxy-21-chloro-1,4-pregnadien-3,20-dione (750 mg, 1.98 mmol) and Cl_2_Ni(PPh_3_)_2 _(35 mg, 0.053 mmol) were added successively. The mixture was stirred at 90°C for 4 h. The TLC showed that the starting material was consumed completely. The mixture was filtered and the filtrate was poured into ice water(150 ml) and extracted with ethyl acetate. The organic layer was washed with brine, dried over Na_2_SO_4_, filtered and concentrated under reduced pressure. The residue was isolated by chromatography on silicagel column with petroleum ether/EtOAc(1:1) as eluent to afford the pure title compound, which was recrystallized from ethyl acetate to give colorless crystals for the single-crystal X-ray diffraction analysis. Yield: 26.5%.

### Refinement

In the absence of significant anomalous scattering, Friedel pairs were merged. All H atoms from C-H groups were positioned geometrically and refined using a riding model with C—H equal 0.93 - 0.98Å and *U*_iso_(H) = 1.2*U*_eq_(C), except methyl groups where *U*_iso_(H) = 1.5*U*_eq_(C). The H atoms of the OH groups were fully refined.

### Crystal data

**Table:**

C_21_H_28_O_4_·H_2_O	*F*(000) = 392
*M**_r_* = 362.45	*D*_x_ = 1.249 Mg m^−^^3^
Monoclinic, *P*2_1_	Mo *K*α radiation, λ = 0.71073 Å
Hall symbol: P 2yb	Cell parameters from 1024 reflections
*a* = 6.641 (2) Å	θ = 2.8–27.1°
*b* = 18.642 (6) Å	µ = 0.09 mm^−^^1^
*c* = 8.017 (3) Å	*T* = 293 K
β = 103.797 (4)°	Plate, colorless
*V* = 963.9 (6) Å^3^	0.25 × 0.20 × 0.15 mm
*Z* = 2	

### Data collection

**Table:**

Bruker SMART APEX CCD area-detector diffractometer	2182 independent reflections
Radiation source: fine-focus sealed tube	1778 reflections with *I* > 2σ(*I*)
graphite	*R*_int_ = 0.034
φ and ω scans	θ_max_ = 27.1°, θ_min_ = 2.8°
Absorption correction: multi-scan (*SADABS*; Sheldrick, 2008a)	*h* = −8→8
*T*_min_ = 0.978, *T*_max_ = 0.987	*k* = −23→23
4866 measured reflections	*l* = −10→6

### Refinement

**Table:**

Refinement on *F*^2^	Primary atom site location: structure-invariant direct methods
Least-squares matrix: full	Secondary atom site location: difference Fourier map
*R*[*F*^2^ > 2σ(*F*^2^)] = 0.042	Hydrogen site location: inferred from neighbouring sites
*wR*(*F*^2^) = 0.104	H atoms treated by a mixture of independent and constrained refinement
*S* = 0.97	*w* = 1/[σ^2^(*F*_o_^2^) + (0.0626*P*)^2^] where *P* = (*F*_o_^2^ + 2*F*_c_^2^)/3
2182 reflections	(Δ/σ)_max_ < 0.001
254 parameters	Δρ_max_ = 0.24 e Å^−^^3^
5 restraints	Δρ_min_ = −0.15 e Å^−^^3^

### Special details

**Table:**

Geometry. All e.s.d.'s (except the e.s.d. in the dihedral angle between two l.s. planes) are estimated using the full covariance matrix. The cell e.s.d.'s are taken into account individually in the estimation of e.s.d.'s in distances, angles and torsion angles; correlations between e.s.d.'s in cell parameters are only used when they are defined by crystal symmetry. An approximate (isotropic) treatment of cell e.s.d.'s is used for estimating e.s.d.'s involving l.s. planes.
Refinement. Refinement of *F*^2^ against ALL reflections. The weighted *R*-factor *wR* and goodness of fit *S* are based on *F*^2^, conventional *R*-factors *R* are based on *F*, with *F* set to zero for negative *F*^2^. The threshold expression of *F*^2^ > σ(*F*^2^) is used only for calculating *R*-factors(gt) *etc*. and is not relevant to the choice of reflections for refinement. *R*-factors based on *F*^2^ are statistically about twice as large as those based on *F*, and *R*- factors based on ALL data will be even larger.

### Fractional atomic coordinates and isotropic or equivalent isotropic displacement parameters (Å^2^)

**Table:**

	*x*	*y*	*z*	*U*_iso_*/*U*_eq_	
O1	0.1926 (4)	0.41488 (11)	0.9326 (3)	0.0555 (5)	
O2	0.6229 (3)	0.61548 (11)	0.4180 (3)	0.0509 (5)	
H2X	0.691 (4)	0.6524 (13)	0.440 (4)	0.055 (10)*	
O3	0.1121 (4)	0.77632 (12)	−0.2435 (3)	0.0583 (6)	
O4	−0.0063 (3)	0.78258 (11)	0.1395 (3)	0.0499 (5)	
H4X	−0.050 (4)	0.8214 (11)	0.102 (4)	0.039 (8)*	
O5	0.8509 (4)	0.74238 (15)	0.4291 (4)	0.0686 (7)	
H5Y	0.936 (5)	0.756 (3)	0.523 (4)	0.089 (15)*	
H5X	0.894 (6)	0.750 (2)	0.340 (4)	0.086 (14)*	
C1	0.4634 (4)	0.52002 (13)	0.6909 (4)	0.0427 (6)	
H1	0.5753	0.5514	0.7111	0.051*	
C2	0.4114 (4)	0.49166 (14)	0.8260 (4)	0.0433 (6)	
H2	0.4894	0.5028	0.9355	0.052*	
C3	0.2351 (4)	0.44350 (13)	0.8073 (4)	0.0409 (6)	
C4	0.1154 (4)	0.43065 (13)	0.6331 (4)	0.0403 (6)	
H4	−0.0030	0.4023	0.6169	0.048*	
C5	0.1682 (4)	0.45781 (13)	0.4951 (3)	0.0371 (6)	
C6	0.0458 (4)	0.44352 (13)	0.3178 (4)	0.0437 (6)	
H6A	−0.0788	0.4173	0.3230	0.052*	
H6B	0.1265	0.4136	0.2591	0.052*	
C7	−0.0148 (4)	0.51213 (14)	0.2155 (4)	0.0418 (6)	
H7A	−0.1214	0.5364	0.2585	0.050*	
H7B	−0.0738	0.4997	0.0964	0.050*	
C8	0.1668 (4)	0.56397 (12)	0.2240 (3)	0.0328 (5)	
H8	0.2664	0.5417	0.1673	0.039*	
C9	0.2742 (4)	0.57722 (12)	0.4142 (3)	0.0332 (5)	
H9	0.1632	0.5932	0.4671	0.040*	
C10	0.3559 (4)	0.50534 (13)	0.5089 (3)	0.0366 (6)	
C11	0.4311 (4)	0.63875 (13)	0.4440 (4)	0.0404 (6)	
H11	0.4551	0.6519	0.5655	0.048*	
C12	0.3482 (4)	0.70560 (12)	0.3407 (3)	0.0392 (6)	
H12A	0.4596	0.7404	0.3542	0.047*	
H12B	0.2406	0.7265	0.3886	0.047*	
C13	0.2592 (4)	0.69250 (12)	0.1481 (3)	0.0337 (5)	
C14	0.0878 (4)	0.63452 (12)	0.1298 (3)	0.0342 (5)	
H14	−0.0177	0.6538	0.1847	0.041*	
C15	−0.0196 (5)	0.62098 (16)	−0.0586 (4)	0.0485 (7)	
H15A	0.0780	0.5987	−0.1153	0.058*	
H15B	−0.1337	0.5879	−0.0644	0.058*	
C16	−0.1023 (5)	0.68968 (17)	−0.1538 (4)	0.0535 (7)	
H16A	−0.1490	0.6796	−0.2756	0.064*	
H16B	−0.2203	0.7067	−0.1135	0.064*	
C17	0.0599 (4)	0.74649 (14)	−0.1266 (4)	0.0415 (6)	
C17A	0.1594 (4)	0.76373 (13)	0.0615 (3)	0.0396 (6)	
C18	0.4297 (4)	0.67001 (16)	0.0598 (4)	0.0453 (6)	
H18A	0.4766	0.6226	0.0963	0.068*	
H18B	0.5437	0.7030	0.0901	0.068*	
H18C	0.3758	0.6704	−0.0625	0.068*	
C19	0.5131 (4)	0.46446 (16)	0.4290 (4)	0.0526 (7)	
H19A	0.5383	0.4178	0.4805	0.079*	
H19B	0.6408	0.4908	0.4493	0.079*	
H19C	0.4575	0.4594	0.3075	0.079*	
C20	0.3116 (5)	0.82621 (15)	0.0757 (4)	0.0559 (8)	
H20A	0.3711	0.8364	0.1946	0.084*	
H20B	0.2395	0.8678	0.0213	0.084*	
H20C	0.4196	0.8136	0.0201	0.084*	

### Atomic displacement parameters (Å^2^)

**Table:**

	*U*^11^	*U*^22^	*U*^33^	*U*^12^	*U*^13^	*U*^23^
O1	0.0786 (14)	0.0499 (11)	0.0436 (12)	−0.0146 (10)	0.0255 (10)	0.0001 (9)
O2	0.0356 (9)	0.0403 (11)	0.0753 (15)	−0.0050 (8)	0.0104 (10)	0.0063 (10)
O3	0.0817 (14)	0.0535 (12)	0.0414 (12)	−0.0120 (11)	0.0181 (10)	0.0069 (10)
O4	0.0664 (12)	0.0407 (10)	0.0477 (12)	0.0179 (9)	0.0239 (10)	0.0110 (9)
O5	0.0723 (15)	0.0808 (17)	0.0542 (15)	−0.0300 (13)	0.0177 (13)	0.0031 (13)
C1	0.0380 (13)	0.0372 (13)	0.0484 (16)	−0.0063 (11)	0.0014 (12)	0.0077 (12)
C2	0.0526 (15)	0.0356 (13)	0.0384 (15)	−0.0026 (11)	0.0040 (12)	0.0039 (11)
C3	0.0528 (14)	0.0294 (12)	0.0445 (16)	0.0016 (11)	0.0195 (12)	0.0001 (11)
C4	0.0419 (13)	0.0328 (12)	0.0482 (16)	−0.0065 (10)	0.0147 (12)	0.0030 (11)
C5	0.0410 (12)	0.0267 (11)	0.0430 (15)	−0.0010 (10)	0.0091 (11)	−0.0008 (10)
C6	0.0512 (14)	0.0335 (13)	0.0460 (15)	−0.0118 (11)	0.0111 (13)	−0.0026 (12)
C7	0.0439 (14)	0.0387 (13)	0.0404 (15)	−0.0111 (11)	0.0053 (12)	−0.0011 (12)
C8	0.0346 (12)	0.0295 (11)	0.0329 (13)	−0.0045 (9)	0.0055 (10)	−0.0011 (9)
C9	0.0339 (11)	0.0309 (11)	0.0345 (13)	−0.0027 (9)	0.0079 (10)	0.0011 (9)
C10	0.0339 (12)	0.0348 (13)	0.0414 (14)	−0.0007 (10)	0.0098 (10)	0.0072 (11)
C11	0.0410 (13)	0.0378 (13)	0.0385 (15)	−0.0083 (10)	0.0017 (11)	0.0030 (11)
C12	0.0460 (13)	0.0309 (12)	0.0385 (14)	−0.0086 (10)	0.0056 (11)	−0.0009 (10)
C13	0.0377 (12)	0.0283 (11)	0.0344 (13)	−0.0044 (9)	0.0073 (10)	−0.0003 (9)
C14	0.0350 (12)	0.0332 (12)	0.0337 (14)	−0.0032 (9)	0.0069 (10)	−0.0012 (10)
C15	0.0567 (16)	0.0434 (14)	0.0386 (15)	−0.0154 (13)	−0.0024 (13)	0.0006 (12)
C16	0.0551 (15)	0.0609 (18)	0.0382 (16)	−0.0120 (14)	−0.0014 (13)	0.0072 (13)
C17	0.0504 (14)	0.0373 (13)	0.0357 (15)	0.0037 (11)	0.0082 (12)	0.0060 (11)
C17A	0.0497 (14)	0.0338 (13)	0.0365 (14)	0.0013 (11)	0.0129 (12)	0.0019 (10)
C18	0.0452 (13)	0.0444 (14)	0.0498 (17)	−0.0029 (12)	0.0180 (12)	−0.0005 (12)
C19	0.0520 (16)	0.0423 (15)	0.068 (2)	0.0121 (13)	0.0239 (15)	0.0125 (14)
C20	0.075 (2)	0.0386 (15)	0.0492 (18)	−0.0124 (14)	0.0058 (15)	0.0027 (13)

### Geometric parameters (Å, °)

**Table:**

O1—C3	1.228 (3)	C9—H9	0.9800
O2—C11	1.407 (3)	C10—C19	1.549 (4)
O2—H2X	0.821 (19)	C11—C12	1.525 (3)
O3—C17	1.209 (4)	C11—H11	0.9800
O4—C17A	1.433 (3)	C12—C13	1.535 (4)
O4—H4X	0.810 (18)	C12—H12A	0.9700
O5—H5Y	0.86 (2)	C12—H12B	0.9700
O5—H5X	0.85 (2)	C13—C18	1.531 (4)
C1—C2	1.323 (4)	C13—C14	1.551 (3)
C1—C10	1.489 (4)	C13—C17A	1.570 (3)
C1—H1	0.9300	C14—C15	1.530 (4)
C2—C3	1.454 (4)	C14—H14	0.9800
C2—H2	0.9300	C15—C16	1.524 (4)
C3—C4	1.452 (4)	C15—H15A	0.9700
C4—C5	1.337 (4)	C15—H15B	0.9700
C4—H4	0.9300	C16—C17	1.489 (4)
C5—C6	1.483 (4)	C16—H16A	0.9700
C5—C10	1.512 (3)	C16—H16B	0.9700
C6—C7	1.521 (4)	C17—C17A	1.529 (4)
C6—H6A	0.9700	C17A—C20	1.528 (4)
C6—H6B	0.9700	C18—H18A	0.9599
C7—C8	1.535 (3)	C18—H18B	0.9599
C7—H7A	0.9700	C18—H18C	0.9599
C7—H7B	0.9700	C19—H19A	0.9599
C8—C9	1.541 (3)	C19—H19B	0.9599
C8—C14	1.545 (3)	C19—H19C	0.9599
C8—H8	0.9800	C20—H20A	0.9599
C9—C11	1.530 (3)	C20—H20B	0.9599
C9—C10	1.571 (3)	C20—H20C	0.9599
			
C11—O2—H2X	101 (2)	C13—C12—H12A	108.6
C17A—O4—H4X	108 (2)	C11—C12—H12B	108.6
H5Y—O5—H5X	114 (4)	C13—C12—H12B	108.6
C2—C1—C10	124.8 (2)	H12A—C12—H12B	107.6
C2—C1—H1	117.6	C18—C13—C12	111.0 (2)
C10—C1—H1	117.6	C18—C13—C14	111.9 (2)
C1—C2—C3	121.5 (3)	C12—C13—C14	107.69 (19)
C1—C2—H2	119.2	C18—C13—C17A	107.9 (2)
C3—C2—H2	119.2	C12—C13—C17A	109.33 (19)
O1—C3—C4	122.3 (2)	C14—C13—C17A	108.94 (19)
O1—C3—C2	121.3 (3)	C15—C14—C8	111.5 (2)
C4—C3—C2	116.3 (2)	C15—C14—C13	111.5 (2)
C5—C4—C3	122.8 (2)	C8—C14—C13	113.00 (19)
C5—C4—H4	118.6	C15—C14—H14	106.8
C3—C4—H4	118.6	C8—C14—H14	106.8
C4—C5—C6	122.2 (2)	C13—C14—H14	106.8
C4—C5—C10	122.4 (2)	C16—C15—C14	112.5 (2)
C6—C5—C10	115.4 (2)	C16—C15—H15A	109.1
C5—C6—C7	112.3 (2)	C14—C15—H15A	109.1
C5—C6—H6A	109.1	C16—C15—H15B	109.1
C7—C6—H6A	109.1	C14—C15—H15B	109.1
C5—C6—H6B	109.1	H15A—C15—H15B	107.8
C7—C6—H6B	109.1	C17—C16—C15	111.3 (2)
H6A—C6—H6B	107.9	C17—C16—H16A	109.4
C6—C7—C8	113.7 (2)	C15—C16—H16A	109.4
C6—C7—H7A	108.8	C17—C16—H16B	109.4
C8—C7—H7A	108.8	C15—C16—H16B	109.4
C6—C7—H7B	108.8	H16A—C16—H16B	108.0
C8—C7—H7B	108.8	O3—C17—C16	123.0 (3)
H7A—C7—H7B	107.7	O3—C17—C17A	122.1 (3)
C7—C8—C9	108.5 (2)	C16—C17—C17A	114.9 (2)
C7—C8—C14	110.02 (19)	O4—C17A—C20	110.1 (2)
C9—C8—C14	111.72 (19)	O4—C17A—C17	106.6 (2)
C7—C8—H8	108.8	C20—C17A—C17	110.9 (2)
C9—C8—H8	108.8	O4—C17A—C13	107.5 (2)
C14—C8—H8	108.8	C20—C17A—C13	114.2 (2)
C11—C9—C8	114.3 (2)	C17—C17A—C13	107.2 (2)
C11—C9—C10	114.83 (19)	C13—C18—H18A	109.5
C8—C9—C10	111.49 (19)	C13—C18—H18B	109.5
C11—C9—H9	105.0	H18A—C18—H18B	109.5
C8—C9—H9	105.0	C13—C18—H18C	109.5
C10—C9—H9	105.0	H18A—C18—H18C	109.5
C1—C10—C5	111.9 (2)	H18B—C18—H18C	109.5
C1—C10—C19	106.4 (2)	C10—C19—H19A	109.5
C5—C10—C19	107.8 (2)	C10—C19—H19B	109.5
C1—C10—C9	110.3 (2)	H19A—C19—H19B	109.5
C5—C10—C9	106.31 (19)	C10—C19—H19C	109.5
C19—C10—C9	114.1 (2)	H19A—C19—H19C	109.5
O2—C11—C12	113.2 (2)	H19B—C19—H19C	109.5
O2—C11—C9	110.6 (2)	C17A—C20—H20A	109.5
C12—C11—C9	112.6 (2)	C17A—C20—H20B	109.5
O2—C11—H11	106.7	H20A—C20—H20B	109.5
C12—C11—H11	106.7	C17A—C20—H20C	109.5
C9—C11—H11	106.7	H20A—C20—H20C	109.5
C11—C12—C13	114.8 (2)	H20B—C20—H20C	109.5
C11—C12—H12A	108.6		
			
C10—C1—C2—C3	−1.7 (4)	O2—C11—C12—C13	74.9 (3)
C1—C2—C3—O1	176.9 (3)	C9—C11—C12—C13	−51.5 (3)
C1—C2—C3—C4	−2.3 (4)	C11—C12—C13—C18	−66.8 (3)
O1—C3—C4—C5	−175.5 (3)	C11—C12—C13—C14	56.0 (3)
C2—C3—C4—C5	3.7 (4)	C11—C12—C13—C17A	174.3 (2)
C3—C4—C5—C6	179.3 (2)	C7—C8—C14—C15	−59.1 (3)
C3—C4—C5—C10	−1.0 (4)	C9—C8—C14—C15	−179.7 (2)
C4—C5—C6—C7	127.3 (3)	C7—C8—C14—C13	174.4 (2)
C10—C5—C6—C7	−52.5 (3)	C9—C8—C14—C13	53.8 (3)
C5—C6—C7—C8	49.2 (3)	C18—C13—C14—C15	−61.1 (3)
C6—C7—C8—C9	−52.6 (3)	C12—C13—C14—C15	176.6 (2)
C6—C7—C8—C14	−175.2 (2)	C17A—C13—C14—C15	58.1 (3)
C7—C8—C9—C11	−168.7 (2)	C18—C13—C14—C8	65.4 (3)
C14—C8—C9—C11	−47.2 (3)	C12—C13—C14—C8	−56.9 (3)
C7—C8—C9—C10	59.0 (2)	C17A—C13—C14—C8	−175.36 (19)
C14—C8—C9—C10	−179.54 (19)	C8—C14—C15—C16	179.1 (2)
C2—C1—C10—C5	4.1 (4)	C13—C14—C15—C16	−53.6 (3)
C2—C1—C10—C19	−113.5 (3)	C14—C15—C16—C17	50.2 (4)
C2—C1—C10—C9	122.3 (3)	C15—C16—C17—O3	124.4 (3)
C4—C5—C10—C1	−2.7 (3)	C15—C16—C17—C17A	−54.6 (4)
C6—C5—C10—C1	177.0 (2)	O3—C17—C17A—O4	125.0 (3)
C4—C5—C10—C19	114.0 (3)	C16—C17—C17A—O4	−56.0 (3)
C6—C5—C10—C19	−66.2 (3)	O3—C17—C17A—C20	5.2 (4)
C4—C5—C10—C9	−123.2 (3)	C16—C17—C17A—C20	−175.8 (3)
C6—C5—C10—C9	56.6 (3)	O3—C17—C17A—C13	−120.1 (3)
C11—C9—C10—C1	46.5 (3)	C16—C17—C17A—C13	58.9 (3)
C8—C9—C10—C1	178.55 (19)	C18—C13—C17A—O4	177.4 (2)
C11—C9—C10—C5	168.0 (2)	C12—C13—C17A—O4	−61.7 (2)
C8—C9—C10—C5	−59.9 (3)	C14—C13—C17A—O4	55.7 (3)
C11—C9—C10—C19	−73.2 (3)	C18—C13—C17A—C20	−60.2 (3)
C8—C9—C10—C19	58.8 (3)	C12—C13—C17A—C20	60.7 (3)
C8—C9—C11—O2	−81.9 (3)	C14—C13—C17A—C20	178.2 (2)
C10—C9—C11—O2	48.8 (3)	C18—C13—C17A—C17	63.1 (3)
C8—C9—C11—C12	45.8 (3)	C12—C13—C17A—C17	−176.0 (2)
C10—C9—C11—C12	176.5 (2)	C14—C13—C17A—C17	−58.6 (3)

### Hydrogen-bond geometry (Å, °)

**Table:**

*D*—H···*A*	*D*—H	H···*A*	*D*···*A*	*D*—H···*A*
O2—H2X···O5	0.82 (2)	2.00 (2)	2.799 (3)	166 (3)
C1—H1···O2	0.93	2.72	3.191 (3)	112
C12—H12A···O5	0.97	2.52	3.315 (4)	139
C18—H18A···O2	0.96	2.53	3.028 (4)	112
C19—H19B···O2	0.96	2.34	2.915 (4)	118
O4—H4X···O1^i^	0.81 (2)	1.97 (2)	2.758 (3)	164 (3)
O5—H5Y···O3^ii^	0.86 (2)	1.99 (2)	2.851 (4)	171 (4)
O5—H5X···O4^iii^	0.85 (2)	1.97 (2)	2.811 (3)	172 (4)
C16—H16A···O5^iv^	0.97	2.64	3.427 (4)	138
C6—H6A···O3^v^	0.97	2.70	3.297 (3)	120

Symmetry codes: (i) −*x*, *y*+1/2, −*z*+1; (ii) *x*+1, *y*, *z*+1; (iii) *x*+1, *y*, *z*; (iv) *x*−1, *y*, *z*−1; (v) −*x*, *y*−1/2, −*z*.
